# Zebrafish Mnx proteins specify one motoneuron subtype and suppress acquisition of interneuron characteristics

**DOI:** 10.1186/1749-8104-7-35

**Published:** 2012-11-05

**Authors:** Steve D Seredick, Liesl Van Ryswyk, Sarah A Hutchinson, Judith S Eisen

**Affiliations:** 1 Institute of Neuroscience, 1254 University of Oregon, Eugene, OR, 97403, USA; 2 Current address: Program in Developmental & Stem Cell Biology, The Hospital for Sick Children, 101 College Street, MaRS East Tower, Room 11-601, Toronto, ON, M5G 1L7, Canada

**Keywords:** Zebrafish, Mnx, Motoneuron, Interneuron

## Abstract

**Background:**

Precise matching between motoneuron subtypes and the muscles they innervate is a prerequisite for normal behavior. Motoneuron subtype identity is specified by the combination of transcription factors expressed by the cell during its differentiation. Here we investigate the roles of Mnx family transcription factors in specifying the subtypes of individually identified zebrafish primary motoneurons.

**Results:**

Zebrafish has three Mnx family members. We show that each of them has a distinct and temporally dynamic expression pattern in each primary motoneuron subtype. We also show that two Mnx family members are expressed in identified VeLD interneurons derived from the same progenitor domain that generates primary motoneurons. Surprisingly, we found that Mnx proteins appear unnecessary for differentiation of VeLD interneurons or the CaP motoneuron subtype. Mnx proteins are, however, required for differentiation of the MiP motoneuron subtype. We previously showed that MiPs require two temporally-distinct phases of Islet1 expression for normal development. Here we show that in the absence of Mnx proteins, the later phase of Islet1 expression is initiated but not sustained, and MiPs become hybrids that co-express morphological and molecular features of motoneurons and V2a interneurons. Unexpectedly, these hybrid MiPs often extend CaP-like axons, and some MiPs appear to be entirely transformed to a CaP morphology.

**Conclusions:**

Our results suggest that Mnx proteins promote MiP subtype identity by suppressing both interneuron development and CaP axon pathfinding. This is, to our knowledge, the first report of transcription factors that act to distinguish CaP and MiP subtype identities. Our results also suggest that MiP motoneurons are more similar to V2 interneurons than are CaP motoneurons.

## Background

The ability of an animal to carry out behavior depends on precise innervation of each muscle by the appropriate motoneuron subtype. Motoneuron subtype identity is specified by the combination of transcription factors expressed by a cell during its differentiation, and recognized by characteristic features, such as soma position, axon trajectory and muscle innervation pattern. Although specification of motoneuron subtype identity has been well-studied [1,2], we still have an incomplete picture of the molecular mechanisms regulating this process. Here we take advantage of the ability to recognize individual primary motoneurons (PMNs) in the spinal cord of embryonic zebrafish to explore the roles of Mnx family transcription factors in motoneuron subtype specification.

Spinal cord neurons develop from distinct progenitor domains defined by expression of specific transcription factors [2,3]. Zebrafish PMNs are derived from the progenitor of motoneuron (pMN) domain [4] and comprise three subtypes: CaP, MiP and RoP, each of which can be distinguished based on soma position, axon trajectory and muscle innervation [5]. A fourth PMN, VaP, is variably present, initially equivalent to CaP, and later typically dies [6,7]. Here we focus on CaP, which innervates ventral myotome and MiP, which innervates dorsal myotome. Initially both CaP and MiP express Islet1, a transcription factor required for PMN development. In the absence of Islet1, PMNs develop axon trajectories and express the neurotransmitter characteristic of VeLD interneurons [8], which are also derived from the pMN domain [4]. Later in development, CaP down-regulates Islet1 and expresses a related protein, Islet2a. MiP also down-regulates Islet1, but then re-expresses it about an hour later [9,10]. The second phase of Islet1 expression is regulated by Nkx6 transcription factors. In the absence of Nkx6 proteins, MiP axon formation begins normally with the extension of a ventral axon to the muscle pioneers, an identified set of muscle fibers that separate dorsal and ventral muscle [11]. However, MiP then fails to extend its normal axon collateral to dorsal muscle, and instead develops an interneuron-like axon within the spinal cord [10]. This interneuron-like axon often resembles axons of V2a interneurons [12,13] that are derived from the p2 domain situated just dorsal to the pMN domain [3]. The p2 domain, which generates excitatory V2a and inhibitory V2b neurons, has been shown to be closely related to the pMN domain based on shared expression of a number of transcription factors [2].

The vertebrate Mnx family comprises homeodomain transcription factors originally isolated in human and subsequently isolated in chick and mouse [14]. Mnx2 (previously called MNR2 and Hlxb9l [14]) was isolated from a single chick cell induced to become a motoneuron [15]. Mnx2 is expressed in motoneuron progenitors and in post-mitotic motoneurons. Ectopic expression of Mnx2 is sufficient to induce motoneuron differentiation in Islet1-positive spinal cord neurons; whether Mnx2 is necessary for motoneuron differentiation has not been tested. Mnx1 (previously called Hb9 and Hlxb9 [14]) was isolated in mice and shown to be necessary for normal differentiation of many motoneurons [16,17]. In its absence, motoneurons still project axons to the periphery, but the axon projections are abnormal and the cells inappropriately express a marker of V2a interneurons [18]. In both chick and mouse, Mnx proteins are exclusive to motoneurons at early stages of spinal cord development [15-17]; however, later in development Mnx1 is expressed in a small set of interneurons [19].

We provide evidence for a novel role of Mnx proteins in zebrafish motoneuron subtype specification. Zebrafish have three Mnx proteins, Mnx1 and two co-orthologs of Mnx2, Mnx2a and Mnx2b [20], all of which are expressed primarily in post-mitotic neurons. We show that each Mnx family member is expressed in a distinct pattern in each PMN subtype, and that this pattern is dynamic during PMN differentiation. In contrast to early developmental stages in chicks and mice when Mnx expression within the spinal cord is exclusive to motoneurons [15-17], two zebrafish Mnx family members are expressed in VeLD interneurons. We used morpholino antisense oligonucleotides (MOs [21]) to knock down Mnx function and found, to our surprise, that CaPs and VeLDs developed normally. In contrast, Mnx proteins are required for normal MiP development. In their absence, the second phase of MiP Islet1 expression is initiated at the appropriate time, but is lost a few hours later. MiPs in *mnx* MO-injected embryos express markers of V2a interneurons, similar to what has been reported in mouse mutants [16,17]. However, in contrast to mouse mutants, these MiPs also developed V2a-like axons in addition to peripheral axons projecting to muscle. Surprisingly, the peripheral axons of these MiPs did not extend to their normal dorsal muscle targets, but instead projected ventrally alongside normal CaP axons. In some cases, MiPs appeared to be entirely transformed to a CaP morphology in the absence of Mnx proteins. These studies identify Mnx proteins as essential in preventing MiPs from expressing characteristics of V2a interneurons. They also reveal an unexpected role for Mnx proteins in specifying MiP subtype identity by preventing MiPs from developing as CaPs.

## Results

### *mnx* family genes are dynamically expressed in primary motoneurons and VeLD interneurons

We characterized expression of *mnx1*, *mnx2a* and *mnx2b* within the zebrafish spinal cord using RNA *in situ* hybridization. To determine the specific PMNs that express each *mnx* gene, we simultaneously labeled *islet1* mRNA, which is initially expressed in all PMNs [9] and each of the *mnx* family members. At later stages we included either *islet1*, which is expressed in RoP and MiP, or *islet2a*, which is expressed in CaP and VaP [9]. After 20 hpf, *islet1* and *islet2a* are also expressed by smaller, more ventrally-located secondary motoneurons [9,22,23] that we excluded from our analyses.

**Figure 1:**
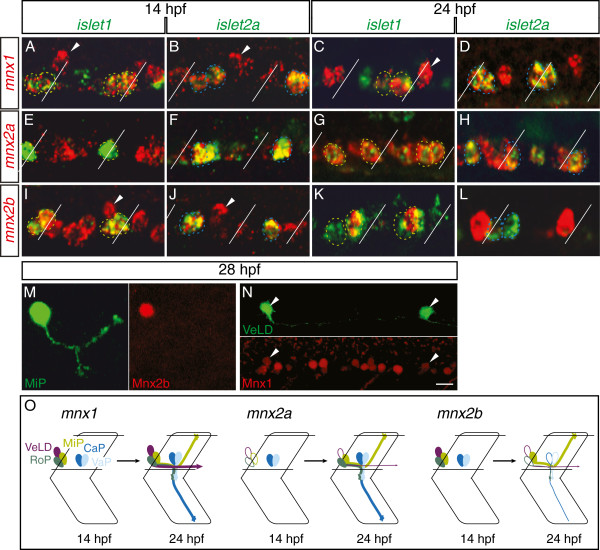
**Zebrafish *mnx *family genes are dynamically expressed in PMNs and VeLD interneurons.** Lateral views, anterior to the left; all figures are in this orientation unless otherwise noted. MiPs and RoPs are outlined green in panels with *islet1* labeling, CaPs and VaPs are outlined blue in panels with *islet2a* labeling. Segment boundaries are demarcated with diagonal lines. (**A**-**L**) RNA expression; two spinal hemisegments are shown in each panel. At 14 hpf, *mnx1* is co-expressed with both *islet1* (**A**) and *islet2a* (**B**), *mnx2a* is only co-expressed with *islet2a* (**E**, **F**), and *mnx2b* is co-expressed with both *islet1* (**I**) and *islet2a* (**J**). The VeLD interneuron can be seen expressing both *mnx1* (arrowheads **A, B**) and *mnx2b* (arrowheads **I, J**). At 24 hpf, *mnx1* maintains co-expression with both *islet1* (**C**) and *islet2a* (**D**), *mnx2a* is co-expressed with both *islet1* (**G**) and *islet2a* (**H**), and *mnx2b* is co-expressed only with *islet1* (**K**, **L**). VeLD still expresses *mnx1* (arrowhead **C**). (**M**-**N**) Protein expression. Mnx2b protein (red) co-localizes with the MiP soma (GFP; green) (**M**). Mnx1 protein (red) co-localizes with the VeLD soma (GFP; green) (**N**). (**O**) Schematic of *mnx* expression. Scale bar: 10 μm, **A**-**L**; 10 μmm M; 20 μm **N**.

Each *mnx* gene has a dynamic and specific expression pattern in each of the PMNs. *mnx1* was expressed in all four PMNs from 14 to 24 hpf (Figure 1A-D). In contrast, *mnx2a* was initially expressed in only CaP and VaP from 14 to 18 hpf (Figure 1E, F). However, by 20 hpf, *mnx2a* expression has expanded to all four PMNs, a pattern that persisted through 24 hpf (Figure 1G, H). *mnx2b* was initially expressed in all four PMNs from 14 to 18 hpf (Figure 1I, J). Intriguingly, by 20 hpf, *mnx2b* expression was reduced to a single *islet1*^+^ PMN (Figure 1K, L). To learn which PMN expressed *mnx2b* after 20 hpf, we injected *UAS*:*GFP* plasmid into *Tg*(*mnx1*:*GAL4*) embryos, which resulted in mosaic expression of GFP and thus revealed both the soma and axon trajectory of GFP-positive neurons. We then processed the embryos at 28 hpf for GFP and Mnx2b immunohistochemistry. Based on its expression of *islet1* at 24 hpf (Figure 1K) and dorsal axon, the Mnx2b^+^ PMN is MiP (Figure 1M).

In addition to expression in PMNs, *mnx1* and *mnx2b* are also expressed in a slightly more dorsal cell first visible at about 14 hpf (Figure 1A, I). *mnx1* expression persisted in this cell through 24 hpf, but *mnx2b* expression was extinguished around 20 hpf. The position and early appearance of these *mnx1*^+^*mnx2b*^+^ cells suggested that they were VeLD interneurons, which can be uniquely identified based on their lateral position, soma shape, and axon trajectory [4,24-26]. To test this, we labeled VeLDs by injecting the *UAS*:*GFP* plasmid into *Tg*(*mnx1*:*GAL4*) embryos and verified that they expressed Mnx1 by immunohistochemistry (Figure 1N). We also showed that *mnx1* is co-expressed with the VeLD marker *gad* (*glutamic acid decarboxylase*, an enzyme in the synthetic pathway for the neurotransmitter GABA; [8,27-29]), but not with *vglut* (*vesicular glutamate transporter*), a marker of excitatory, glutamatergic V2a interneurons [12] (Additional file 1: Figure S1).

To explore the possibility that *mnx* genes are expressed by other interneurons with descending axons we looked for co-expression of *mnx1* and *vsx2* or *gata3*, markers of V2a and V2b fate, respectively [13,29]. Expression of *mnx1* and these markers was always mutually exclusive (Additional file 1: Figure S1 and data not shown), ruling out expression of *mnx* family genes in V2a and V2b interneurons. Based on its descending axon, early appearance and *gad* expression, the interneuron positive for expression of both *mnx1* and *mnx2b* is VeLD.

In mice and chicks, members of the *mnx* gene family are expressed in motoneuron progenitors prior to exit from the cell cycle. In zebrafish, PMNs and VeLDs adjacent to somites 5 to 15 emerge from *olig2*:*GFP*^+^ progenitors in the pMN domain [4], exit the cell cycle between 9 and 16 hpf [30], and then down-regulate *olig2*[31], although GFP persists for a short time. To determine if zebrafish Mnx proteins are expressed in PMN progenitors, we examined expression in *Tg*(*olig2*:*GFP*) embryos (Additional file 2: Figure S2). We found Mnx1^+^ cells that were either GFP^-^ or expressed low levels of GFP; these cells often co-expressed Elavl3, a marker of post-mitotic neurons but did not co-express phosphohistone H3 (PH3), a marker of mitotic cells. Similarly, Mnx2a was often co-expressed with Elavl3 and never co-expressed with PH3, even though Mnx2a was expressed in some cells with high levels of GFP. Although *mnx2b* RNA was present as early as 14 hpf, we could not detect Mnx2b protein until at least 20 hpf, and then it was present only in MiPs (Figure 1O). Together these data are most consistent with the idea that all three Mnx proteins are first expressed in post-mitotic neurons and that expression of Mnx2a precedes expression of Mnx1.

In addition to examining Mnx expression in early-developing PMNs and VeLD interneurons, we also characterized Mnx expression in later-developing secondary motoneurons (SMNs). By 26 hpf, Mnx1 is expressed in a subset of mostly dorsally-located SMNs (Additional file 3: Figure S3), Mnx2a is expressed in a subset of mostly ventrally-located SMNs (Additional file 3: Figure S3), and Mnx2b is rarely expressed in SMNs (data not shown).

### *mnx* expression is independent of Islet1

Islet1 and Lhx3 cooperate to regulate Mnx1 expression in chicks [32,33]. To learn whether this relationship is conserved in zebrafish, we injected MOs to knock down either Islet1 [8], or Lhx3 and Lhx4 (Hutchinson SA, Seredick S, Van Ryswyk L, Talbot JC, Eisen JS: Lhx3 and Lhx4 regulate interneuron fate and prevent motoneurons from co-expressing interneuron characteristics, unpublished), and examined *mnx* gene expression. Surprisingly, expression of all three *mnx* genes was unaffected by Islet1 knockdown (Additional file 4: Figure S4). Moreover, at 24 hpf only *mnx2b* expression was eliminated in the absence of Lhx3 and Lhx4, revealing that Islet1 and Lhx3 do not cooperate to regulate *mnx* expression in zebrafish (Additional file 4: Figure S4).

Overexpression of Mnx proteins in chicks induces formation of ectopic motoneurons expressing Islet, Lhx3 and other Mnx paralogs [15,34]. To test the hypothesis that Mnx proteins regulate expression of *lhx* genes in zebrafish, we injected *mnx* MOs and examined expression of *lhx3* and *lhx4*. We found that neither *lhx3* nor *lhx4* expression was affected by the absence of Mnx proteins. We also eliminated expression of Mnx1, Mnx2a and Mnx2b individually and examined expression of each *mnx* gene. In the absence of any one *mnx* gene, expression of other paralogs was unaffected, revealing that each member of the gene family is regulated independently of the others (Additional file 4: Figure S4).

### Mnx proteins are unnecessary for formation of primary motoneurons and VeLD interneurons

**Figure 2:**
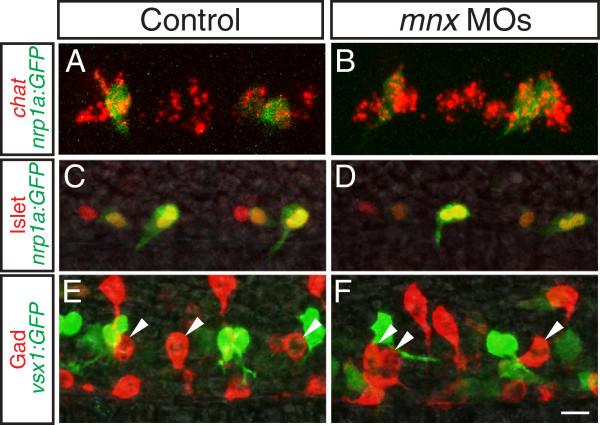
**Mnx proteins are not required for formation of PMNs or VeLDs.** Control and *mnx* MO-injected embryos labeled with markers of PMN or VeLD identity. (**A**-**B**) At 16 hpf, PMNs express *chat* and *nrp1a*:*GFP* in control (**A**) and *mnx* MO-injected embryos (**B**). (**C**-**D**) At 18 hpf, PMNs express Islet in control (**C**) and *mnx* MO-injected *Tg*(*nrp1a*:*GFP*) embryos (**D**). (**E**-**F**) At 20 hpf, VeLD interneuron (arrowheads) numbers and distribution are the same in control (**E**) and *mnx* MO-injected *Tg*(*vsx1*:*GFP*) embryos (**F**); *n* = 80 segments in 10 embryos for control and *mnx* MO-injected samples. Scale bar: 15 μm, A-B; 20 μm, **C-F**.

To test the function of Mnx proteins in PMN development we used previously validated translation-blocking morpholinos [20] (Additional file 5: Figure S5). To determine whether PMNs form in the absence of Mnx proteins, we assessed three markers of PMN identity: Islet [8], *chat*, which encodes an enzyme required to synthesize acetylcholine [35] and *nrp1a*:*GFP*, a transgene expressed in CaP and VaP before 18 hpf [36] and in all PMNs at later stages. All three markers were expressed normally in the combined absence of Mnx1, Mnx2a, and Mnx2b (Figure 2A-D), indicating that PMN specification is normal.

To assess whether VeLD development was compromised in the absence of Mnx proteins, we examined expression of Gad65/67, the biosynthetic enzyme for GABA, in *Tg*(*vsx1*:*GFP*) embryos. VeLDs express Gad65/67 but not *vsx1*:*GFP*, and can be uniquely identified by their lateral position and soma shape. At 20 hpf, the number of VeLDs in the absence of all three Mnx proteins was indistinguishable from controls (Figure 2E, F). Moreover, at 28 hpf VeLDs were morphologically normal in the absence of Mnx proteins (data not shown). Together these data provide evidence that Mnx proteins are not required for VeLD or PMN generation, and that both cell types acquire aspects of their mature identity in the absence of Mnx proteins.

### Mnx proteins promote formation of normal MiP axons

**Figure 3:**
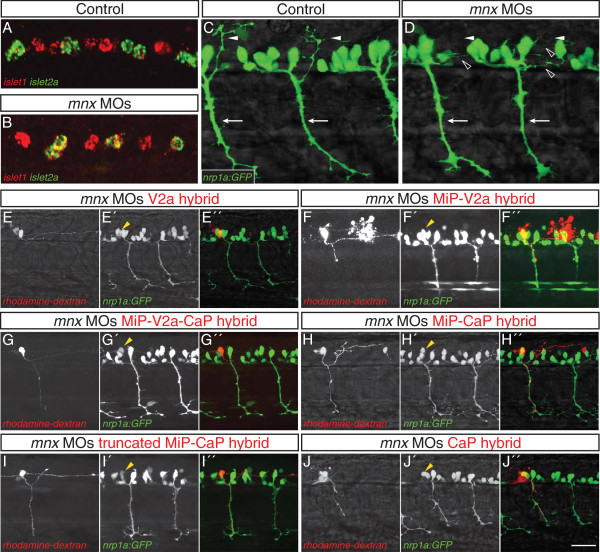
**Mnx proteins promote MiP subtype identity and prevent interneuron**-**like and CaP**-**like processes.** (**A**-**D**) Control and *mnx* MO-injected embryos. (**A**) In controls *islet1* and *islet2a* expression is mutually exclusive at 18 hpf. (**B**) In embryos injected with *mnx1*, *mnx2a*, and *mnx2b* MOs, *islet1* is co-expressed with *islet2a* in CaP and VaP. (**C**) Control embryos have normal, ventrally-projecting CaP axons (arrows) and dorsally-projecting MiP axons (arrowheads). (**D**) CaP axons (arrows) are normal in *mnx* MO-injected embryos whereas MiP axons (closed arrowheads) are absent and there are ectopic interneuron-like axons (open arrowheads). (**E**-**J**^′′^) Rhodamine-dextran-labeled MiPs in 28 to 32 hpf *Tg*(*nrp1a*:*GFP*) embryos injected with *mnx1*, *mnx2a*, and *mnx2b* MOs. Panels show rhodamine-dextran labeling (no superscript), *nrp1a*:*GFP* (^′^) and a merged image of the two channels (^′′^). Labeled MiPs (yellow arrowheads) become hybrids with six morphologies. (**E**-**E**^′′^) V2a hybrids express *nrp1a*:*GFP* and have a descending V2a-like axon. (**F**-**F**^′′^) MiP-V2a hybrids have a descending V2a-like axon as well as a normal-appearing MiP ventral axon; the red blob to the right is a cell that was killed during labeling. (**G**-**G**^′′^) MiP-V2a-CaP hybrids have a descending V2a-like axon as well as a ventrally-projecting CaP-like axon. (**H**-**H**^′′^) MiP-CaP hybrids have both a normal MiP axon and a CaP-like axon. (**I**-**I**^′′^) Truncated MiP-CaP hybrids have a truncated MiP dorsal axon as well as a ventrally-projecting CaP-like axon. (**J**-**J**^′′^) CaP hybrids have a CaP-like axon that extends next to a normal CaP axon. Scale bar: 20 μm, A-D; 40 μm, **E-H**^′′^.

Because *mnx* genes are expressed during and after the period of PMN subtype commitment [25], we examined whether Mnx proteins play a role in subtype specification. Normally all PMNs express *islet1* as they exit the cell cycle and then later express only one *islet* gene characteristic of their subtype: MiP and RoP express *islet1*, whereas CaP and VaP express *islet2a*[9]. In the absence of all three Mnx proteins, CaPs inappropriately expressed both *islet1* and *islet2a* (Figure 3A, B). However, these cells formed normal, ventrally-extending CaP axons and appeared morphologically indistinguishable from CaPs in control embryos (Figure 3C, D), consistent with our previous finding that *islet1* and *islet2a* can play equivalent roles in CaP specification [8].

Strikingly, MiP dorsal axons were almost entirely absent from embryos lacking all three Mnx proteins (Figure 3C, D; Table 1). To determine if a subset of the Mnx proteins is responsible for the MiP axon phenotype, we knocked down each Mnx protein singly or in pairs and counted the number of MiP axons in the mid-trunk. We saw no phenotype in the absence of any single Mnx protein, or in the absence of Mnx1 plus Mnx2a, or Mnx1 plus Mnx2b. However, in the absence of both Mnx2a and Mnx2b, MiP axons were absent from nearly half the segments, and when present they were often truncated (Table 1). Thus, the two Mnx2 paralogs appear to play a predominant role in formation of normal, dorsally-projecting MiP axons, although the increased severity of the triple Mnx protein knockdown demonstrates that all three Mnx proteins are involved in this process.

**Table 1:** Mnx proteins are required for MiP formation

	**CaP axons Normal**	**Normal**	**MiP axons Truncated**	**Absent**
**Control**	**100**%	**97**%	**1**%	**1**%
	*n* = 30	*n* = 85	*n* = 85	*n* = 85
	10 embryos	18 embryos	18 embryos	18 embryos
***mnx2a*** +	**100**%	**26**%	**25**%	**49**%
***mnx2b*MOs**	*n* = 85	*n* = 85	*n* = 85	*n* = 85
	18 embryos	18 embryos	18 embryos	18 embryos
***mnx1*** +	**95**%	**11**%	**13**%	**76**%
***mnx2a*** +	*n* = 87	*n* = 87	*n* = 87	*n* = 87
***mnx2b*MOs**	19 embryos	19 embryos	19 embryos	19 embryos

Assayed at 28 to 32 hpf, segments 8 to 12 of *Tg*(*nrp1a*:*GFP*).

*n*, number of segments.

**Figure 4:**
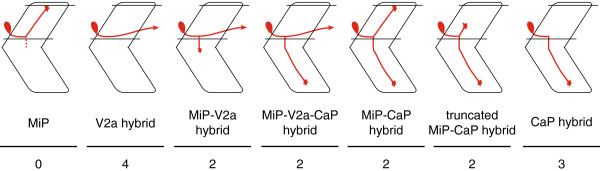
**Mnx proteins prevent MiPs from acquiring inappropriate axonal projections.** In the absence of Mnx proteins, MiPs adopt one of 6 alternative hybrid axonal projections. The number of labeled MiPs we observed in each category is indicated below the diagram of each alternative hybrid.

The absence of normal MiP dorsal axons from *mnx* MO-injected embryos led us to consider whether MiPs developed abnormal axon projections. Consistent with this idea, *nrp1a*:*GFP*^+^ descending interneuron axons were present in the ventral spinal cords of embryos lacking all three Mnx proteins, something never seen in control embryos (Figure 3C, D). To examine the morphology of MiPs in triple *mnx* MO-injected *Tg*(*nrp1a*:*GFP*) embryos in more detail, we labeled individual GFP-expressing cells in the MiP position with rhodamine-dextran. During these experiments, we noted that unlike CaPs, which express GFP very brightly in triple *mnx* MO-injected *Tg*(*nrp1a*:*GFP*) embryos, MiPs were difficult to detect because they expressed GFP dimly, if at all. When we scored GFP^+^, rhodamine-labeled MiPs, we observed a range of phenotypes. In some cases we labeled GFP^+^ cells that had only a V2a interneuron-like axon that descended many segments within the spinal cord (V2a hybrid; Figure 3E-E”; Figure 4). Some MiPs initiated motoneuron development by projecting a normal-appearing ventral axon that stopped at the muscle pioneers. Instead of also projecting a collateral to dorsal muscle, however, these cells developed a V2a interneuron-like axon (MiP-V2a hybrid; Figure 3F-F”; Figure 4). Surprisingly, in some cases the ventral axons of labeled MiPs failed to stop at the muscle pioneers, instead extending as far ventrally as CaP axons (MiP-V2a-CaP hybrid; Figure 3G-G”; Figure 4). In other cases, labeled cells had both a normal MiP axon and a CaP-like axon (MiP-CaP hybrid; Figure 3H-H”; Figure 4), or had both a truncated MiP axon and a CaP-like axon (truncated MiP-CaP hybrid; Figure 3I-I”; Figure 4). We also labeled MiPs that had only a CaP-like axon (CaP hybrid; Figure J-J”; Figure 4), suggesting that some MiPs entirely transformed to a CaP morphology. Based on these observations, we conclude that Mnx proteins are required both to prevent MiPs from developing V2a interneuron-like axons and to prevent MiPs from extending a ventral axon along the pathway normally followed by the CaP axon.

### Mnx proteins prevent MiPs from acquiring molecular characteristics of V2a interneurons

**Figure 5:**
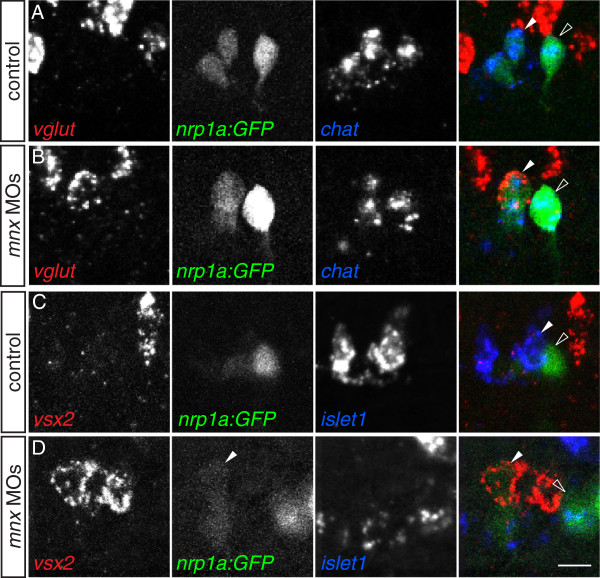
**Mnx proteins prevent MiPs from acquiring V2a**-**like molecular characteristics.** (**A**-**D**) Single spinal hemisegments of control and *mnx* MO-injected embryos. MiPs (closed arrowheads) and CaPs (open arrowheads) are indicated in merged panels (right column). (**A**) In control embryos, MiPs and CaPs express *chat* but never *vglut* (0/20 *vglut*^+^ MiPs or CaPs in five embryos). (**B**) In *mnx* MO-injected embryos, MiPs co-express both *chat* and *vglut* (22/41 *vglut*^+^ MiPs in eight embryos), while CaPs express *chat* but not *vglut* (0/41 *vglut*^+^ CaPs in eight embryos. (**C**) In control embryos, MiPs express *islet1* but not *vsx2* (0/50 *vsx2*^+^ MiPs in 10 embryos), while CaPs express neither *islet1* nor *vsx2* (0/50 *vsx2*^+^ CaPs in 10 embryos). (**D**) In *mnx* MO-injected embryos, MiP express *vsx2* but not *islet1* (26/60 *vsx2*^+^ MiPs in 13 embryos) while CaPs express neither *vsx2* nor *islet1* (0/60 *vsx2*^+^ CaPs in 13 embryos). Scale bar: 10 μm.

To ascertain whether MiPs in *mnx*-deficient embryos take on molecular as well as morphological characteristics of V2a interneurons, we assayed for co-expression of interneuron and motoneuron markers. We found that in triple *mnx* MO-injected embryos, MiPs co-expressed cholinergic and glutamatergic markers (Figure 5A, B). This phenotype was never seen in MiPs or CaPs in control embryos or in CaPs in *mnx* MO-injected embryos. This hybrid neurotransmitter phenotype is specific, as expression of cholinergic and GABAergic or glycinergic markers was always mutually exclusive in both controls and MO-injected embryos (Additional file 6: Figure S6).

Although V2a interneurons are the only cells in the ventral spinal cord that express glutamatergic markers before 32 hpf [37], we also examined expression of *vsx2*, a definitive V2a marker [13]. In 22 hpf control *Tg*(*nrp1a*:*GFP*) embryos, we found 1.2 *vsx2*^+^ cells per spinal hemisegment. In controls, expression of *vsx2* and the motoneuron markers *islet1* and *nrp1a*:GFP was mutually exclusive (Figure 5C). In the absence of Mnx proteins, we found 2.3 *vsx2*^+^ cells per spinal hemisegment. Often, the extra *vsx2*^+^ cell weakly expressed GFP and was located near the somite boundary (Figure 5D), in the position occupied by the MiP soma, suggesting the hybrid cells expressing both PMN and interneuron markers were MiPs. By comparison, *vsx2* was never expressed in CaPs, which continued to express *islet2a* in the absence of Mnx proteins (Figures 3B and 5D). These results suggest that Mnx proteins act to block expression of V2a interneuron markers specifically within MiPs.

### Mnx proteins regulate MiP axon formation by maintaining Islet1 expression

Previously we found that in the absence of Nkx6.1 and Nkx6.2, most MiPs failed to form dorsal axons, and instead projected both their normal short ventral axon to the muscle pioneers and an interneuron-like axon within the spinal cord [10]. The similarity of the Nkx6 and Mnx knockdown phenotypes suggested that the genes might be part of the same pathway. To test this hypothesis, we injected *nkx6* MOs and examined Mnx expression, and we injected *mnx* MOs and examined Nkx6 expression. We found that *mnx* expression was unaffected by the absence of Nkx6 proteins (Additional file 4: Figure S4). Similarly, *nkx6* expression was unaffected by the absence of all three Mnx proteins (Figure 5). This indicates that Mnx proteins influence MiP development independently of Nkx6.

**Figure 6:**
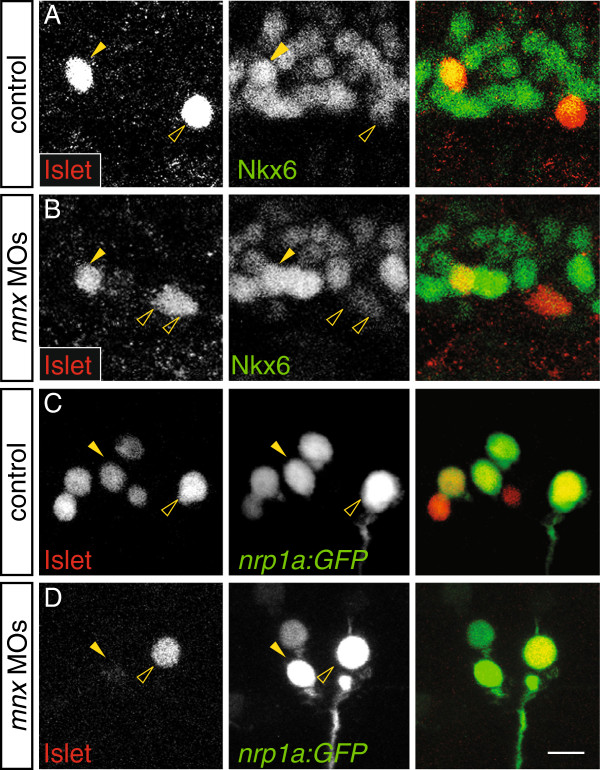
**Mnx proteins maintain the late phase of Islet1 expression in MiPs independently of Nkx6.** (**A**-**D**) Single spinal hemisegments of control and *mnx* MO-injected embryos. MiPs (closed arrowheads) and CaPs (open arrowheads) are indicated in each of the single channel panels; right column shows merged channels. (**A**) At 17 hpf, MiPs in control embryos strongly express Islet and Nkx6 (17/30 Nkx6^+^ MiPs in seven embryos). CaPs strongly express Islet but weakly express Nkx6. (**B**) MiPs in *mnx* MO-injected embryos continue to strongly express Islet and Nkx6 (13/22 Nkx6^+^ MiPs in five embryos). (**C**) At 21 hpf in *Tg*(*nrp1a*:*GFP*) control embryos, both MiPs and CaPs express Islet (32/32 Islet^+^ CaPs, and 31/32 Islet^+^ in five embryos. (**D**) In *mnx* MO-injected *Tg*(*nrp1a*:*GFP*) embryos, CaPs (60/60 Islet^+^ CaPs in 10 embryos) but not MiPs (19/60 Islet^+^ MiPs in 10 embryos), strongly express Islet. Scale bar: 20 μm.

Nkx6 proteins initiate a late, MiP-specific phase of Islet1 expression [10]. The MiP dorsal axon phenotype was rescued by co-injection of *islet1* mRNA with the *nkx6* MOs, demonstrating that it is this late phase of Islet1 that is required for MiPs to form a normal dorsal axon [10]. To learn whether the MiP axon phenotype in *mnx* MO-injected embryos also depended on the late phase of Islet1 expression, we examined whether this second phase of Islet1 was appropriately expressed in the absence of Mnx proteins. Expression of Islet1 at 18 hpf was normal in the absence of Mnx proteins (Figure 2); however, by 21 hpf Islet1 expression in MiPs was either absent or barely detectable (Figure 6). This suggests that the second phase of Islet1 expression in MiP is initiated correctly in the absence of Mnx proteins, but that Mnx proteins are necessary to maintain expression of Islet1 in MiP, and that continued Islet1 expression is necessary for MiP to form a normal dorsal axon.

## Discussion

**Figure 7:**
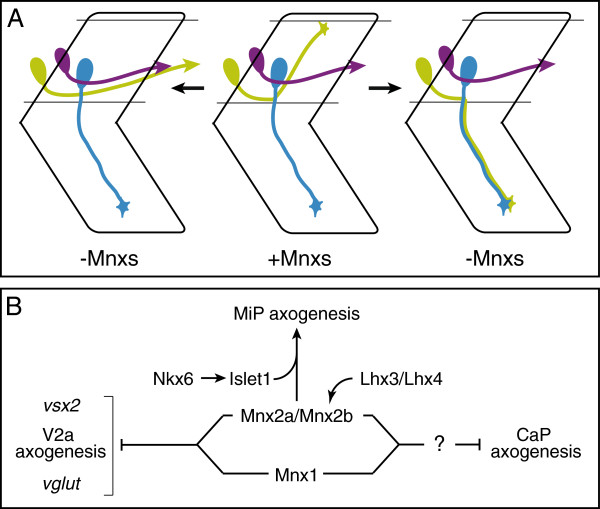
**Mnx proteins promote normal MiP development and suppress acquisition of V2a interneuron and CaP characteristics.** (**A**) Model depicting principal MiP (green) projection errors observed in the absence of Mnx proteins. MiPs often fail to project to their normal dorsal muscle targets and, instead, either project a V2a interneuron-like axon or a motor axon that projects alongside the CaP axon to ventral muscle. Combinations of these three projection errors account for the observed range of MiP phenotypes. By comparison, CaPs (blue) and V2a INs (purple) make normal projections. (**B**) Genetic pathways that account for observed MiP phenotypes. Mnx proteins, primarily Mnx2a and Mnx2b, promote MiP axogenesis by maintaining the late phase of Islet1 expression initiated by Nkx6. All three Mnx proteins (bracket) suppress acquisition of molecular and morphological features of V2a interneurons, and responsiveness to unknown signals that promote axon growth to ventral muscle.

We show that the three Mnx transcription factors have dynamic expression patterns in each of the zebrafish PMN subtypes and in VeLD interneurons. Surprisingly, however, Mnx proteins appear dispensable for development of CaP motoneurons and VeLD interneurons. In contrast, Mnx proteins are required for normal specification of MiP motoneurons through regulation of both axon pathfinding and neurotransmitter specificity (Figure 7).

### Mnx expression in interneurons

Spinal cord expression of *mnx* genes was originally thought to be restricted to motoneurons [15-17]. However, the *Drosophila melanogaster mnx* paralog, *hb9*, is expressed in both motoneurons and interneurons [38,39]. More recently, a small population of Mnx1-expressing interneurons was identified in the mouse spinal cord [19]. Here we identify a class of ventral spinal interneurons in zebrafish, VeLDs, that express two *mnx* genes, *mnx1* and *mnx2b*. The Mnx1-expressing interneurons in mouse are active components in the locomotor central pattern generator [19,40,41]. Although these cells have been extensively characterized during fictive locomotion in isolated spinal cord preparations [42-47], their exact role in the locomotor network in intact animals is unknown. Indeed, mouse Mnx1^+^ interneurons have proven resistant to genetic analysis, in part because their developmental provenance is entirely unclear.

It seems unlikely that VeLDs are the zebrafish equivalent of mouse Mnx1^+^ spinal interneurons. VeLDs are born early, GABA^+^ and have ipsilateral axons that descend many segments within the spinal cord [4,8,27]. In contrast, although the Mnx1^+^ mouse interneurons may arise from the same domain as motoneurons [3], they are likely to be born later than motoneurons as they have not been described in lineage studies. Mouse Mnx1^+^ interneurons are glutamatergic and likely make strictly local projections to motoneuron pools within the same segment [42,46]. We have also noticed some ventromedially-located Mnx1^+^ interneurons that appear at about three days post-fertilization and do not seem to make projections to adjacent segments. Given the striking parallels between well-characterized components of the locomotor network in zebrafish and mouse [12,48], it will be important to follow development of these Mnx1^+^ interneurons *in vivo* to learn their origins. Assessing their role in zebrafish motor behavior should have implications for understanding the contribution of Mnx1^+^ interneurons to locomotion in other vertebrate species.

Despite expression in VeLDs, Mnx proteins appear unnecessary for VeLD development. However, we only assayed axon projection and neurotransmitter phenotype, thus our results do not rule out a role for Mnx proteins in regulating some other aspect of VeLD differentiation.

### Mnx proteins promote MiP subtype identity

The acquisition of MiP and CaP subtype identities are differentially affected by the absence of Mnx1, Mnx2a and Mnx2b. In the absence of all three Mnx proteins, CaPs fail to down-regulate *islet1* expression. However, CaP subtype identity appears unaffected as both axon projection and neurotransmitter expression are normal. This finding is consistent with our previous studies showing that *islet1* and *islet2a* can play equivalent roles in CaP specification [8]. In contrast, a late, MiP-specific phase of *islet1* expression is misregulated in the absence of Mnx proteins. In the absence of high levels of Islet1, MiPs fail to form their characteristic dorsal axons. This is reminiscent of the phenotype observed in the absence of Nkx6 [10], but whereas Nkx6 proteins are required to initiate the late phase of *islet1* expression in MiP, Mnx proteins seem to be required to maintain high levels of Islet1 in MiP, similar to what has been reported in a mouse *Mnx1* knock out [16,17].

Variability in the amount of Islet1 protein or the precise time at which it is cleared might account for variability in MiP morphologies in the absence of Mnx proteins. One possibility is that MiPs that maintain Islet1 expression relatively late retain sufficient motoneuron character to project an axon out of the spinal cord. In contrast, those that down-regulate Islet1 relatively early might fail to express factors necessary to guide growth cones out of the spinal cord and into the periphery. This is consistent with the finding that early expression of Islet1 in CaP is sufficient to permit axon growth into the periphery [8], and could be tested in future experiments with photo-activatable morpholinos [49] to block *islet1* translation at different times and assess the frequency with which MiP axons exit the spinal cord.

Surprisingly, when MiPs in *mnx* MO-injected embryos project axons into the periphery, most of them aberrantly extend to ventral muscle along a pathway normally reserved for CaP axons. Notably, MiP ventral axons stop normally at the muscle pioneers in the absence of Nkx6 proteins and late-phase Islet1 expression [10]. Thus, while Mnx proteins promote formation of dorsal MiP axons by maintaining Islet1 expression, they exclude MiP axons from ventral muscle independently of Islet1.

PMN subtype identity, as revealed by axon trajectory, is influenced by positional signals that normally act during a specific window of developmental time [25,50]. These signals affect motoneuron subtype, at least in part, by regulating expression of factors involved in axon pathfinding. Motor axons navigate toward their appropriate muscle targets by following subtype-specific guidance cues. The cues that are differentially recognized by CaP axons and MiP axons are unknown. Our results suggest that Mnx proteins regulate expression of receptors that recognize cues that prevent the MiP growth cone from progressing ventral of the muscle pioneers, and thus prevent MiPs from becoming CaP-like. To our knowledge, this is the first report of genes that can cause MiPs to transform to a CaP morphology.

### Mnx proteins prevent MiP from acquiring V2a interneuron characteristics

In the absence of Mnx proteins, MiPs, but not CaPs, often form hybrids that have features of both motoneurons and interneurons. Based on expression of *vsx2* and *vglut* and axon morphology, MiPs appear to have acquired features of zebrafish V2a interneurons [12]. As this phenotype is only observed in the combined absence of Mnx1, Mnx2a and Mnx2b, the three zebrafish paralogs act redundantly to suppress the formation of MiP-V2a hybrids. The acquisition of V2a features is reminiscent of the phenotype of *Mnx1* knockout mice in which Vsx2 is inappropriately expressed in a subset of Islet1^+^ motoneurons [16,17]. However, MN-V2a hybrids in *Mnx1* knockout mice fail to project interneuron-like axons within the spinal cord and whether they express glutamatergic markers has not been assessed. Our results suggest that zebrafish MiP-interneuron hybrids acquire a more complete set of V2a features. Regardless, our results reveal a conserved role for the *mnx* gene family in segregating motoneuron from V2a interneuron cell fate in specific motoneuron subtypes.

We previously reported that knocking down Islet1 resulted in PMNs developing as interneurons and that knocking down the Met receptor tyrosine kinase resulted in PMNs co-expressing motoneuron and interneuron characteristics [8,35]. In both of these cases, PMNs expressed the neurotransmitter, GABA. Clonal analysis in zebrafish has revealed that PMNs can be siblings with either KA’ or VeLD interneurons [4], both of which express GABA [4,27]. These observations supported a model whereby many factors expressed by PMNs cooperate to suppress acquisition of characteristics of closely-related interneurons derived from the pMN domain. Here we show that in the absence of Mnx proteins, PMN-interneuron hybrids inappropriately express V2a interneuron characteristics. These data support a model whereby post-mitotic Mnx expression in PMNs suppresses acquisition of characteristics of more distantly-related interneurons from the adjacent p2 domain. A striking aspect of the PMN-V2a phenotype is that it is limited to MiP. This suggests that MiPs are more similar to V2a interneurons than are CaPs. This is consistent with the observation that in the absence of Nkx6 proteins, many MiP-interneuron hybrids have axons with a V2a morphology [10]. However, it is important to note that in our previous Nkx6 knockdown studies, MiPs did not express *vsx2*[10]. As Mnx proteins suppress *vsx2* expression in MiPs (Figure 5), we suspect their continued expression in the absence of Nkx6 (Additional file 4: Figure S4) accounts for our inability to detect *vsx2* expression in MiP in our previous Nkx6 knockdown studies. These combined results imply that other genes in addition to *vsx2*, genes not regulated by Mnx proteins, contribute to acquisition of V2a interneuron axonal projections.

V2as, like PMNs, originate from a domain that expresses Nkx6.1 [12], and continue to express Lhx3 after they exit the cell cycle [29]. Moreover, recent lineage-tracing in mouse has shown that many V2a neurons have expressed *Olig2* during their developmental history [51,52], revealing that they may be even more similar to motoneurons than had been previously appreciated. A more detailed lineage analysis in zebrafish of the relationship between PMNs, VeLD interneurons and V2a neurons could help resolve the relationships among these neurons.

## Conclusions

The three zebrafish Mnx transcription factors have distinct expression patterns in each of the zebrafish PMN subtypes and in VeLD interneurons. These expression patterns are dynamic during the period when these cells are extending axons and initiating neurotransmitter expression. Despite their expression in CaP motoneurons and VeLD interneurons, Mnx proteins appear dispensable for development of these two cell types. In contrast, Mnx proteins are required for development of MiP motoneurons. In the absence of Mnx proteins, MiPs extend aberrant axons and express an interneuron-specific neurotransmitter (Figure 7).

## Methods

### Zebrafish

Wild-type (AB), *Tg*(*olig2*:*GFP*)^*vu12*^[53], *Tg*(*nrp1a*:*GFP*)^*js12*^[36] and *Tg*(*vsx1*:*GFP*)^*nns5*^[13] and *Tg*(*mnx1*:*GFP*)^*ml2*^[54] zebrafish were maintained in a laboratory breeding colony according to established protocols [55]. Embryos collected from natural crosses were allowed to develop at 28.5°C, and staged by hours post-fertilization (hpf) according to morphological criteria [56].

### Generation of transgenic fish lines

A 3-kb fragment of the *mnx1* promoter [54] was subcloned into *p5EMCS*[57]. Multi-site Gateway® technology (Life Technologies; Eugene, OR, USA) was used to assemble an *mnx1*:*GAL4VP16*:*pA* construct flanked by *Tol2* terminal inverted repeats. *Tg*(*mnx1*:*GAL4VP16*) lines were generated by co-injecting plasmid DNA and *Tol2 transposase* RNA [58] into the yolk of one-cell stage embryos. Multiple founders were recovered and characterized; *Tg*(*mnx1*:*GAL4VP16*)^*b1222*^ was chosen for this study because transgene expression faithfully mirrored endogenous Mnx1 protein expression (data not shown).

### Morpholino injections

Approximately 2.5 nl of 100 μM translation-blocking morpholinos (Gene Tools, LLC; Philomath, OR, USA) against *mnx1* (5’-ACCTCACAAACAGATTAACGCCTCG-3’), *mnx2a* (5’-ACCTCACAAACAGATTAACGCCTCG-3’) and *mnx2b* (5’-GACTTTTCCATTGCAACACTTTTGT-3’) were injected into one- to two-cell stage embryos; this was sufficient to suppress translation as assayed by whole-mount immunohistochemistry (Additional file 5: Figure S5) without elevated cell death as assayed by acridine orange staining (data not shown). These morpholinos have been previously validated [20].

Other previously validated morpholinos used in this study include: random control oligonucleotide (5’-N_25_-3’), 2.5 nl of 100 μM *islet1* E2 (5’-TTAATCTGCGTTACCTGATGTAGTC-3’) plus 100 μM *islet1* E3 (5’-GAATGCAATGCCTACCTGCCATTTG-3’) [8] to knockdown *islet1*; 2.5 nl of 400 μM *nkx6*.*1* (5’-CGCAAGAAGAAGGACAGTGACCCG-3’) [59] plus 400 μM *nkx6*.*2* (5’-CGCGCAAAACTCACCCGCACAGGGA-3’) [10] to knockdown *nkx6*.*1* and *nkx6*.*2*; and 2.5 nl of 280 μM *lhx3* (5’-GTTCTAACAACATTCTGGCGATAAA-3’) plus 280 μM *lhx4* (5’-GCAGCACAGCCGCACTTTGCATCAT-3’) to knockdown *lhx3* plus *lhx4* (Hutchinson SA, Seredick S, Van Ryswyk L, Talbot JC, Eisen JS: Lhx3 and Lhx4 regulate interneuron fate and prevent motoneurons from co-expressing interneuron characteristics, unpublished). Morpholino effectiveness was verified by whole mount immunohistochemisry.

### Fluorescent RNA *in situ* hybridization

RNA *in situ* hybridization was performed according to standard protocols [60] with the following modifications. For two-color fluorescent *in situ* hybridization anti-sense probes were labeled with digoxigenin-UTP (Roche Applied Sciences, Indianapolis, IN, USA) and dinitrophenol-UTP (Perkin-Elmer, Waltham, MA, USA). Following overnight hybridization, unbound probe was removed with three 30-minute washes at 68°C in 50% formamide, 5x SSC and 0.1% SDS, followed by stringent washes in 50% formamide, 2x SSC and 0.1% Tween-20. Labeled probes were detected with HRP-conjugated anti-DIG (1:2,000; Jackson ImmunoResearch, West Grove, PA, USA) or HRP-conjugated anti-DNP (1:2,000; Perkin-Elmer) and stained with fluorescein, Cy3- or Cy5-tyramide (1:100; Perkin-Elmer) for 1 to 10 minutes.

Probes used include *mnx1*, *mnx2a* and *mnx2b*[20]; *chat*[35]; *islet1*, *islet2a* and *lhx3*[9]; *lhx4* (Hutchinson SA, Seredick S, Van Ryswyk L, Talbot JC, Eisen JS: Lhx3 and Lhx4 regulate interneuron fate and prevent motoneurons from co-expressing interneuron characteristics, unpublished).; *gad1b* and *gad2* (collectively referred to as *gad*), *slc17a6a*, *slc17a6b* and *slc17a7* (collectively referred to as *vglut*) and *slc6a9* and *slc6a5* (collectively referred to as *glyt*) [28]; *vsx2*[12]; and *gata3*[29].

### Antibody generation

To prepare Mnx1 and Mnx2b antisera, cDNAs corresponding to amino acids 245 to 311 of Mnx1 or amino acids 224 to 301 of Mnx2b were His-tagged, over-expressed in *E*. *coli* and purified by nickel column chromatography under native conditions. These regions are C-terminal to the homeodomain, and are the most divergent regions of the gene family. Purified recombinant proteins were used to immunize rabbits, and the resulting antisera screened by whole mount immunohistochemistry. Attempts to generate antisera against Mnx2a were not successful.

### Immunohistochemistry

Embryos were fixed for 2 hours in 4% paraformaldehyde and 1x Fix Buffer [55] at 4°C, and then treated with 0.5% Triton X-100 in 1x PBS for 15 minutes at room temperature. Embryos were blocked in 5% normal goat serum, 2.5% DMSO and 0.1% Tween-20 in 1x PBS before overnight incubation in diluted primary antibody at 4°C. Unbound primary antibodies were removed by washing for two hours in 1x PBS plus 0.1% Tween-20, followed by overnight incubation in diluted secondary antibody at 4°C. Anti-Mnx1, anti-Mnx2a and anti-Mnx2b were detected with HRP-conjugated goat anti-rabbit and stained with fluorescein-, Cy3- or Cy5-tyramide (1:100; Perkin-Elmer) for one minute; all other primary antibodies were detected with dye-labeled secondary antibodies.

Antibodies used include rabbit polyclonal anti-Mnx1 (1:1,000) and anti-Mnx2b (1:1,000), anti-Mnx2a (1:1,000; AnaSpec, Fremont, CA, USA), anti-Lhx3 and anti-Lhx4 (Hutchinson SA, Seredick S, Van Ryswyk L, Talbot JC, Eisen JS: Lhx3 and Lhx4 regulate interneuron fate and prevent motoneurons from co-expressing interneuron characteristics, unpublished), mouse monoclonal anti-Elavl3/4 (1:10,000; A21271, Life Technologies), anti-Gad (1:500; ab11070, Abcam, Cambridge, MA, USA), anti-GFP (JL-8; Clontech, Mountain View, CA, USA; or A-11120, Life Technologies), anti-Histone H3 (phospho S10; 1:1000; ab14955, Abcam), anti-Islet (39.4D5; DSHB, Iowa City, IA, USA), and anti-Nkx6.1 (F55A10; DSHB).

### Subtype-specific cell labeling

To correlate cell morphology with gene expression, we injected *UAS*:*EGFP* plasmid with *Tol2 transposase* RNA and selected embryos with GFP-expressing cells for immunohistochemistry. Since our *mnx1* morpholino also suppressed expression from our *Tg*(*mnx1*:*GAL4VP16*) transgene, individual neurons in *mnx* morpholino-injected fish were dye-labeled with 5% tetramethylrhodamine-dextran (D-3308; Life Technologies) in 0.2 M KCl [61].

### Image acquisition

All images were acquired on a Zeiss Pascal confocal microscope (Carl Zeiss Microscopy, LLC, Thornwood, New York, USA) using a 40x water immersion objective. The brightness and contrast of images was adjusted using Photoshop CS5 (Version 12.0, Adobe Systems, Inc., San Jose, CA, USA).

### Quantification

All observations of PMNs were made in the mid-trunk region of the spinal cord adjacent to somites 8 to 12. We examined at least 30 segments from 10 embryos for each condition, unless otherwise noted in the figure legends.

## Abbreviations

CaP: Caudal primary motoneuron; KA: Kolmer-Agduhr; Lhx: LIM homeobox; MiP: Middle primary motoneuron; Mnx: Motor neuron and pancreas homeobox; p2: Progenitor2; PMN: Primary motoneuron; pMN: Progenitor of motoneuron; RoP: Rostral primary motoneuron; VeLD: Ventral lateral descending.

## Competing interests

The authors declare that they have no competing interests.

## Authors’ contributions

SS and LVR participated in the design of the study, carried out molecular experiments, and helped draft the manuscript. SAH generated Mnx antibodies and helped draft the manuscript. JSE conceived of the study, labeled individual neurons, and helped draft the manuscript. All authors read and approved the final manuscript.

## Supplementary Material

Additional file 1 **Figure S1.***mnx1* and *mnx2b* are both expressed in VeLD interneurons. (**A**-**D**) VeLD somata are outlined. (**A**) At 16 hpf, *mnx1* and *mnx2b* are co-expressed in VeLD. (**B**) At 24 hpf, *mnx1*^+^ VeLDs express *gad*. (**C**, **D**) At 24 hpf *mnx1*+ VeLDs express neither *vsx2* (**C**) nor *vglut* (**D**). Scale bar: 10 μm. Click here for file

Additional file 2 **Figure S2.** Mnx proteins are restricted to post-mitotic neurons. Lateral views of 12 to 14 hpf *Tg*(*olig2*:*GFP*) embryos. (**A**) Mnx2a^+^ cells within the spinal cord do not co-express phosphohistone H3, a marker of mitotic cells (0/153 Mnx2a^+^ cells in 13 embryos). Some Mnx2a^+^ cells strongly express GFP. (**B**) Mnx1^+^ cells within the spinal cord do not co-express phosphohistone H3 (0/70 Mnx1^+^ cells in 10 embryos). No Mnx1^+^ cells strongly express GFP. (**C**) Mnx2a^+^ cells that expressed GFP weakly or were GFP^-^ co-expressed Elavl3, a marker of post-mitotic neurons. Mnx2a^+^ cells that expressed GFP strongly did not co-express Elavl3. (**D**) Mnx1^+^ cells co-expressed Elavl3. (**E**) Schematic of gene expression during transition from pMN progenitors to post-mitotic neurons. Mitotic progenitors express phosphohistone H3 (PH3), whereas post-mitotic neurons express Elavl3. *olig2* expression is initiated in progenitors, and down-regulated as cells become post-mitotic. Both Mnx1 and Mnx2a expression is initiated after cells become postmitotic, with expression of Mnx2a preceding expression of Mnx1. Scale bar: 30 μm, **A**-**D**. Click here for file

Additional file 3 **Figure S3.** Mnx proteins are differentially expressed in secondary motoneurons. (**A**-**D**) Protein expression in two spinal hemisegments of 26 hpf *Tg*(*mnx1*:*GFP*) embryos. The single channel panels show Mnx protein expression. (**A**, **B**) Mnx1 is strongly expressed by PMNs and VeLD interneurons, and more weakly expressed by a subset of mostly dorsally-located secondary motoneurons. (**C**, **D**) Mnx2a appears to be down-regulated in PMNs and is expressed by a subset of mostly ventrally-located SMNs. Scale bar: 20 μm. Click here for file

Additional file 4 **Figure S4.** With the exception of *mnx2b* which is regulated by Lhx3 and Lhx4, expression of *mnx* genes is independent of Islet, Nkx6, Lhx3, Lhx4, and other Mnx paralogs. (**AAA**) Lateral views of control and MO-injected embryos colabeled with *islet2a* (green) to mark CaP and VaP. Segment boundaries are demarcated with diagonal lines. At 18 hpf, expression of *mnx1* (**A**, **B**), *mnx2a* (**C**, **D**) and *mnx2b* (**E**, **F**) are unaffected by absence of Islet1. Note that *islet2a* is not expressed in the absence of *islet1*[8]. At 18 hpf, expression of *mnx1* (**A**, **G**), *mnx2a* (**C**, **H**) and *mnx2b* (**E**, **I**) are unaffected by absence of Nkx6.1 and Nkx6.2. At 24 hpf, expression of *mnx1* (**J**, **K**) and *mnx2a* (**L**, **M**) are unaffected by absence of Lhx3 and Lhx4. *mnx2b* is not expressed in the absence of Lhx3 and Lhx4 (**N**, **O**). At 18 hpf, expression of *lhx3* (**P**, **Q**) and *lhx4* (**R**, **S**) are unaffected by absence of Mnx proteins. At 16 hpf, expression of *mnx2a* (**T**, **U**) and *mnx2b* (**V**, **W**) are unaffected by absence of Mnx1. (**XAA**) Embryos labeled only for expression of *mnx* genes. Expression of *mnx1* (**X**, **Y**) and *mnx2b* (**Z**, **AA**) are unaffected by absence of Mnx2a. We did not examine expression of *mnx1* and *mnx2a* in the absence of Mnx2b as we did not detect Mnx2b protein before 20 hpf. Note that panels M, O and Q are reproduced from Figure 1 to facilitate comparison of gene expression in control and MO-injected embryos. Scale bar: 10 μm. Click here for file

Additional file 5 **Figure S5.** Morpholinos targeting *mnx* family genes are specific and effective in knocking down protein. Lateral views of two spinal hemisegments, segment boundaries denoted by diagonal lines, of uninjected and control MO-injected *Tg*(*mnx1*:*GFP*) embryos labeled for antibodies against Mnx1 (**A**, **D**), Mnx2a (**B**, **E**), and Mnx2b (**C**, **F**). Embryos injected with *mnx1* MO lack Mnx1 antibody labeling (**G**), but maintain Mnx2a (**H**) and Mnx2b (**I**) antibody labeling. Embryos injected with *mnx2a* MO lack Mnx2a antibody labeling (**K**), but maintain Mnx1 (**J**) and Mnx2b (**L**) antibody labeling. Embryos injected with *mnx2b* MO lack Mnx2b antibody labeling (**O**), but maintain Mnx1 (**M**) and Mnx2a (**N**) antibody labeling. Scale bar: 20 μm. Click here for file

Additional file 6 **Figure S6.** In the absence of Mnx proteins, neither MiPs nor CaPs aberrantly express GABAergic or glycinergic markers. (**A**-**D**) Lateral views of single hemisegments of control and *mnx* MO-injected embryos. MiPs (closed arrowheads) and CaPs (open arrowheads) are indicated in merged panels (column on right). (**A**, **B**) In control and MO-injected embryos, MiP and CaP express *chat* but not *gads*. (**C**, **D**) In control and MO-injected embryos, MiP and CaP express *chat* but not *glyts*. Scale bar: 20 μm. Click here for file
