# Supplementary figures and images for: Zebrafish Mnx proteins specify one motoneuron subtype and suppress acquisition of interneuron characteristics
Source: Neural Dev. 2012 Nov 5;7:35. doi: 10.1186/1749-8104-7-35 (PMC3570319; doi:10.1186/1749-8104-7-35)

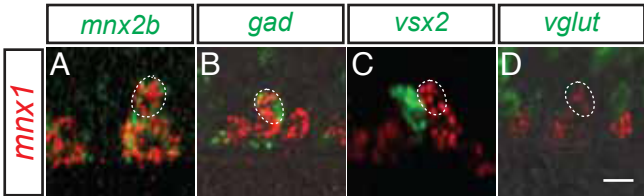

Supplement: Additional file 1 — Figure S1.mnx1 and mnx2b are both expressed in VeLD interneurons. (A-D) VeLD somata are outlined. (A) At 16 hpf, mnx1 and mnx2b are co-expressed in VeLD. (B) At 24 hpf, mnx1+ VeLDs express gad. (C, D) At 24 hpf mnx1+ VeLDs express neither vsx2 (C) nor vglut (D). Scale bar: 10 μm. [file 1749-8104-7-35-S1.pdf]

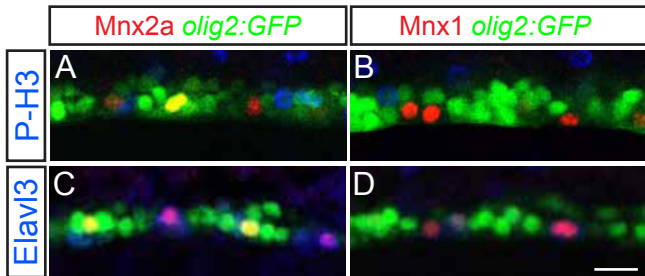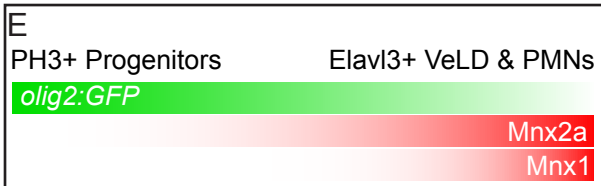

Supplement: Additional file 2 — Figure S2. Mnx proteins are restricted to post-mitotic neurons. Lateral views of 12 to 14 hpf Tg(olig2:GFP) embryos. (A) Mnx2a+ cells within the spinal cord do not co-express phosphohistone H3, a marker of mitotic cells (0/153 Mnx2a+ cells in 13 embryos). Some Mnx2a+ cells strongly express GFP. (B) Mnx1+ cells within the spinal cord do not co-express phosphohistone H3 (0/70 Mnx1+ cells in 10 embryos). No Mnx1+ cells strongly express GFP. (C) Mnx2a+ cells that expressed GFP weakly or were GFP- co-expressed Elavl3, a marker of post-mitotic neurons. Mnx2a+ cells that expressed GFP strongly did not co-express Elavl3. (D) Mnx1+ cells co-expressed Elavl3. (E) Schematic of gene expression during transition from pMN progenitors to post-mitotic neurons. Mitotic progenitors express phosphohistone H3 (PH3), whereas post-mitotic neurons express Elavl3. olig2 expression is initiated in progenitors, and down-regulated as cells become post-mitotic. Both Mnx1 and Mnx2a expression is initiated after cells become postmitotic, with expression of Mnx2a preceding expression of Mnx1. Scale bar: 30 μm, A-D. [file 1749-8104-7-35-S2.pdf]

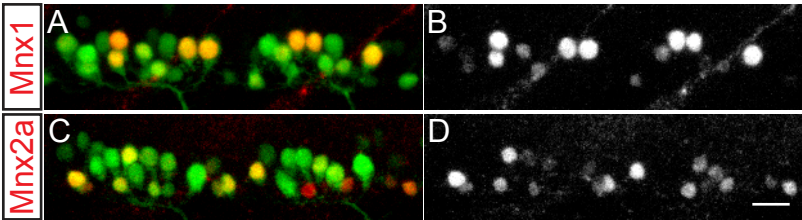

Supplement: Additional file 3 — Figure S3. Mnx proteins are differentially expressed in secondary motoneurons. (A-D) Protein expression in two spinal hemisegments of 26 hpf Tg(mnx1:GFP) embryos. The single channel panels show Mnx protein expression. (A, B) Mnx1 is strongly expressed by PMNs and VeLD interneurons, and more weakly expressed by a subset of mostly dorsally-located secondary motoneurons. (C, D) Mnx2a appears to be down-regulated in PMNs and is expressed by a subset of mostly ventrally-located SMNs. Scale bar: 20 μm. [file 1749-8104-7-35-S3.pdf]

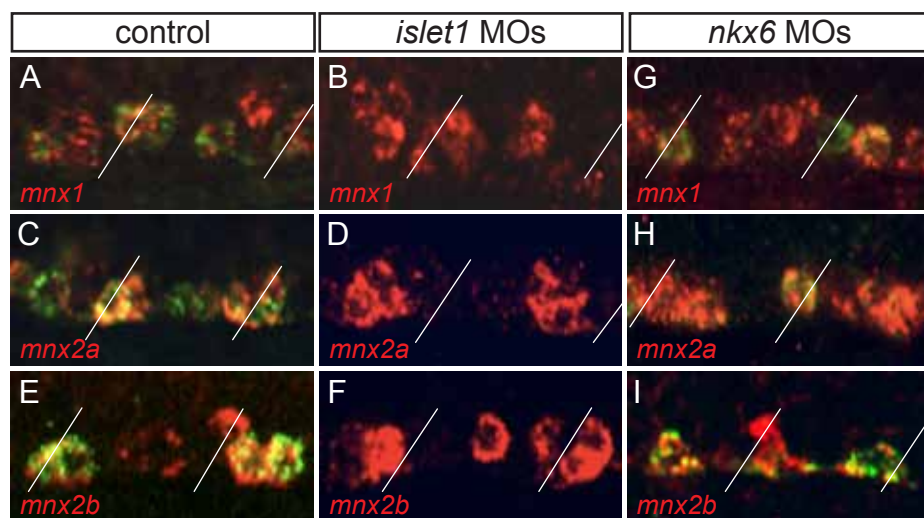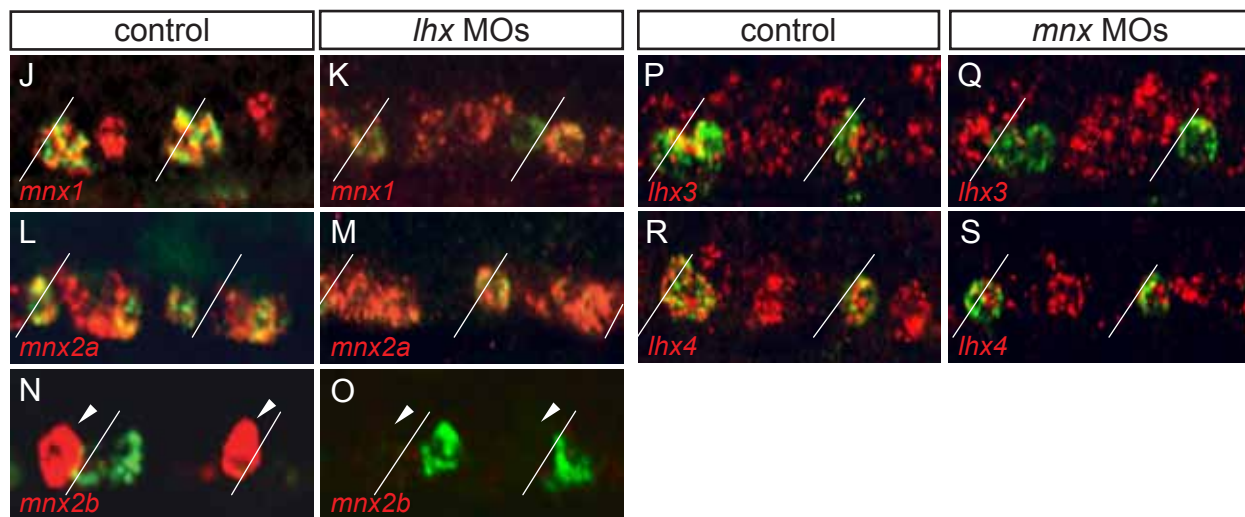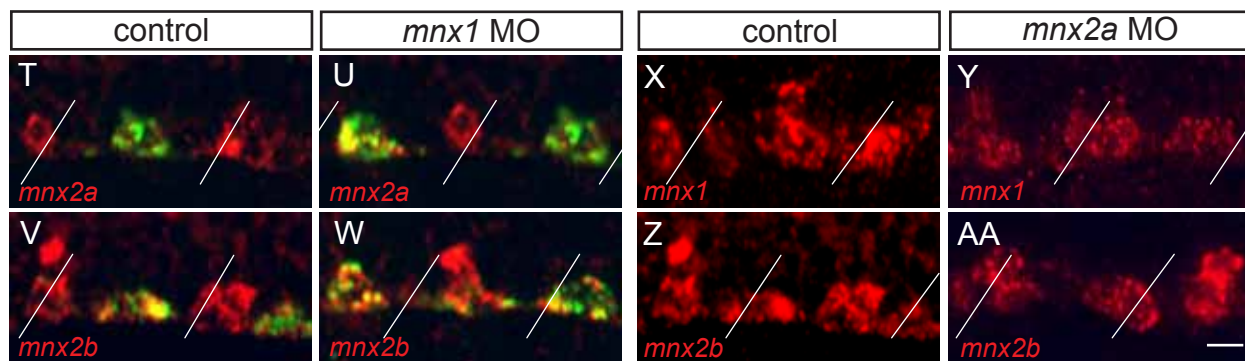

Supplement: Additional file 4 — Figure S4. With the exception of mnx2b which is regulated by Lhx3 and Lhx4, expression of mnx genes is independent of Islet, Nkx6, Lhx3, Lhx4, and other Mnx paralogs. (AAA) Lateral views of control and MO-injected embryos colabeled with islet2a (green) to mark CaP and VaP. Segment boundaries are demarcated with diagonal lines. At 18 hpf, expression of mnx1 (A, B), mnx2a (C, D) and mnx2b (E, F) are unaffected by absence of Islet1. Note that islet2a is not expressed in the absence of islet1[8]. At 18 hpf, expression of mnx1 (A, G), mnx2a (C, H) and mnx2b (E, I) are unaffected by absence of Nkx6.1 and Nkx6.2. At 24 hpf, expression of mnx1 (J, K) and mnx2a (L, M) are unaffected by absence of Lhx3 and Lhx4. mnx2b is not expressed in the absence of Lhx3 and Lhx4 (N, O). At 18 hpf, expression of lhx3 (P, Q) and lhx4 (R, S) are unaffected by absence of Mnx proteins. At 16 hpf, expression of mnx2a (T, U) and mnx2b (V, W) are unaffected by absence of Mnx1. (XAA) Embryos labeled only for expression of mnx genes. Expression of mnx1 (X, Y) and mnx2b (Z, AA) are unaffected by absence of Mnx2a. We did not examine expression of mnx1 and mnx2a in the absence of Mnx2b as we did not detect Mnx2b protein before 20 hpf. Note that panels M, O and Q are reproduced from Figure 1 to facilitate comparison of gene expression in control and MO-injected embryos. Scale bar: 10 μm. [file 1749-8104-7-35-S4.pdf]

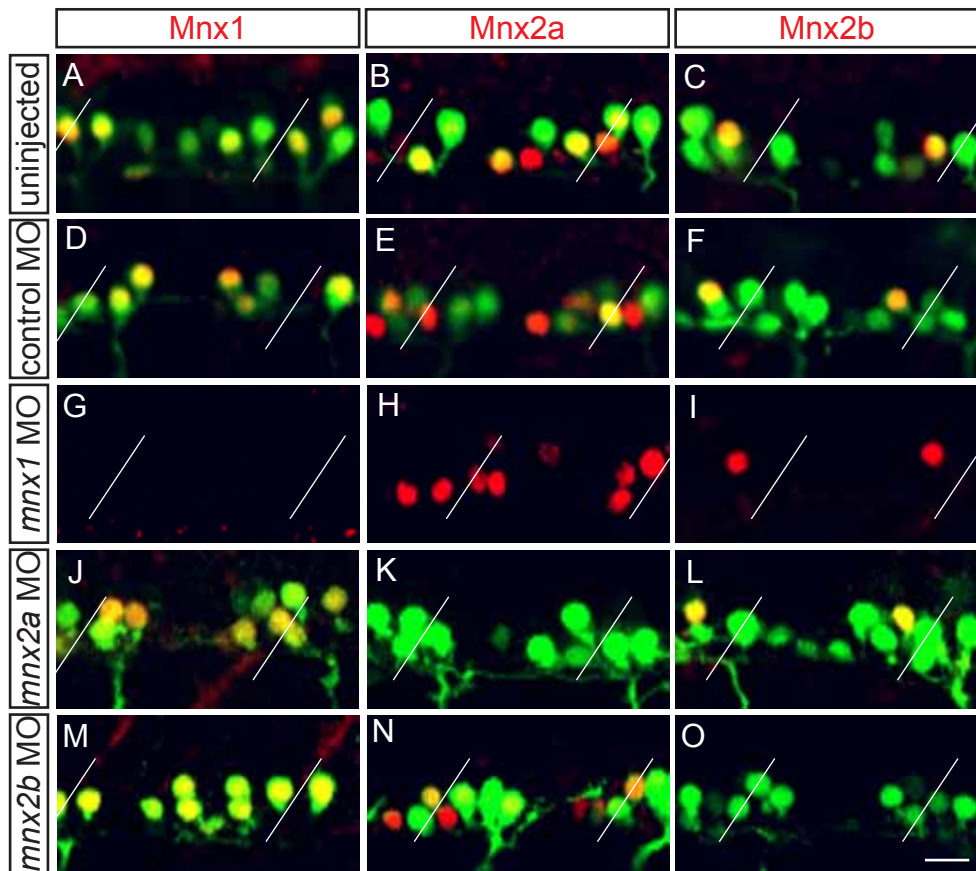

Supplement: Additional file 5 — Figure S5. Morpholinos targeting mnx family genes are specific and effective in knocking down protein. Lateral views of two spinal hemisegments, segment boundaries denoted by diagonal lines, of uninjected and control MO-injected Tg(mnx1:GFP) embryos labeled for antibodies against Mnx1 (A, D), Mnx2a (B, E), and Mnx2b (C, F). Embryos injected with mnx1 MO lack Mnx1 antibody labeling (G), but maintain Mnx2a (H) and Mnx2b (I) antibody labeling. Embryos injected with mnx2a MO lack Mnx2a antibody labeling (K), but maintain Mnx1 (J) and Mnx2b (L) antibody labeling. Embryos injected with mnx2b MO lack Mnx2b antibody labeling (O), but maintain Mnx1 (M) and Mnx2a (N) antibody labeling. Scale bar: 20 μm. [file 1749-8104-7-35-S5.pdf]

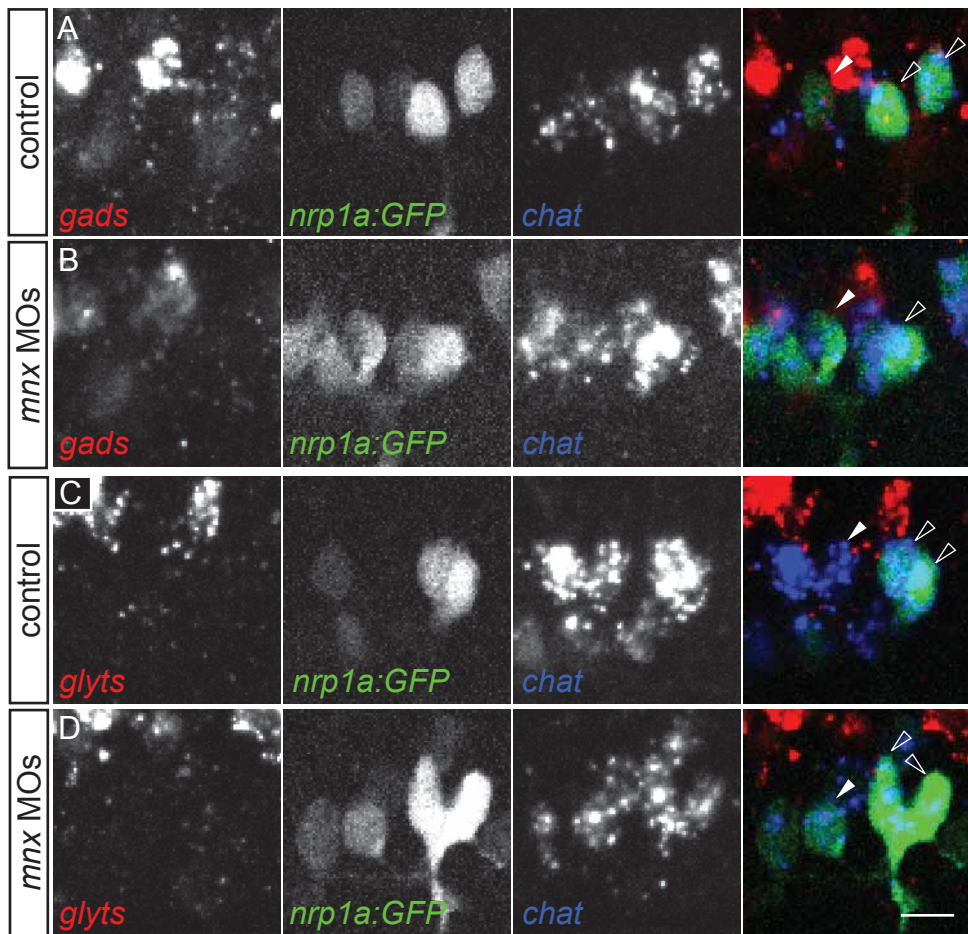

Supplement: Additional file 6 — Figure S6. In the absence of Mnx proteins, neither MiPs nor CaPs aberrantly express GABAergic or glycinergic markers. (A-D) Lateral views of single hemisegments of control and mnx MO-injected embryos. MiPs (closed arrowheads) and CaPs (open arrowheads) are indicated in merged panels (column on right). (A, B) In control and MO-injected embryos, MiP and CaP express chat but not gads. (C, D) In control and MO-injected embryos, MiP and CaP express chat but not glyts. Scale bar: 20 μm. [file 1749-8104-7-35-S6.pdf]
